# Impact of Boiling on Cyanogenic Detoxification and Nutrient Retention in *Cnidoscolus aconitifolius* (Chaya) Leaves

**DOI:** 10.3390/foods15112028

**Published:** 2026-06-05

**Authors:** Chavisa Praditukrit, Chawanphat Muangnoi, Pasitta Panritdum, Jintana Sirivarasai

**Affiliations:** 1 Master of Science Program in Nutrition, Faculty of Medicine Ramathibodi Hospital and Institute of Nutrition, Mahidol University, Bangkok 10400, Thailand; chavimint@gmail.com; 2 Biological Science and Animal Model Unit, Institute of Nutrition, Mahidol University, Nakhon Pathom 73170, Thailand; chawanphat.mua@mahidol.ac.th (C.M.); pasitta15@gmail.com (P.P.); 3 Nutrition Unit, Faculty of Medicine Ramathibodi Hospital, Mahidol University, Bangkok 10400, Thailand

**Keywords:** *Cnidoscolus aconitifolius*, chaya, boiling, cyanogenic compounds, carotenoids, antioxidant capacity, nutrient retention, nutrient retention index

## Abstract

Cyanogenic glycosides in leafy vegetables pose significant food safety concerns because they release hydrogen cyanide (HCN) when plant tissue is disrupted. Although boiling is widely used for detoxification, its effects on nutritional quality and bioactive compounds remain insufficiently characterized. This study evaluated the effects of boiling on HCN, carotenoids, antioxidant capacity, and nutrient retention in *Cnidoscolus aconitifolius* (chaya) leaves. Antioxidant capacity was determined using the oxygen radical absorbance capacity, ferric reducing antioxidant power, and 2,2-diphenyl-1-picrylhydrazyl assays. An integrative nutrient retention index (NRI) was used to assess overall nutrient preservation. Boiling reduced HCN by 99.94%, confirming effective detoxification through hydrolysis, volatilization, and leaching. β-carotene showed high retention (95.8%), whereas thiamine (0.91%) and riboflavin (16.67%) were markedly reduced. Potassium retention was also low (24.85%). The total phenolic content and antioxidant capacity decreased significantly after boiling. The NRI indicated high retention of bioactive compounds (85.95%) but lower retention of vitamins (25.81%) and minerals (52.85%), yielding a global value of 54.92%. These findings highlight a trade-off between food safety and nutritional quality. Therefore, boiling remains a critical safety intervention for chaya and provides a useful model for optimizing processing conditions to balance detoxification with functional value.

## 1. Introduction

Phytochemicals are key bioactive constituents of plant-based foods that contribute to the prevention of chronic diseases, including cardiovascular disorders, metabolic syndrome, and neurodegenerative conditions. Their biological effects are mediated through multiple mechanisms, such as antioxidant activity, modulation of inflammatory pathways, and metabolic regulation [1,2]. Green leafy vegetables are particularly important sources of these compounds, notably polyphenols and carotenoids, which contribute to both nutritional quality and functional health benefits [3,4].

*Cnidoscolus aconitifolius* (chaya) is a traditional leafy vegetable widely consumed in several regions, including Thailand, and is increasingly recognized for its high nutritional density and functional potential [5]. Chaya leaves contain substantial amounts of proteins, minerals, and bioactive compounds, including phenolic acids and carotenoids, which are associated with antioxidant and metabolic benefits [6,7,8]. A single-arm clinical study involving 30 individuals with dyslipidemia reported that daily consumption of 500 mL of chaya beverage for six weeks significantly reduced serum triglycerides and oxidative stress markers while enhancing antioxidant activity. These findings suggest that chaya may improve dyslipidemia, oxidative stress and has potential as a functional food for metabolic health benefits [9].

Despite these benefits, the consumption of chaya is constrained by the presence of cyanogenic glycosides, which release hydrogen cyanide (HCN) upon tissue disruption. This represents a critical food-safety concern, as exposure to HCN can have toxic effects [10,11]. Previous research has documented the cyanogenic glycoside content ranging from 0.79 to 14.79 μg HCN equivalent per gram of fresh weight, with linamarin identified as the predominant cyanogenic compound present in edible Chaya leaves [12]. While there are limited cases of documented human poisoning, toxicological studies involving Wistar rats have indicated an increase in cyanide-related biomarkers and oxidative stress subsequent to Chaya consumption [13]. These findings underscore the potential toxicity risks linked to improper preparation. Consequently, it is strongly recommended that adequate thermal processing, particularly boiling, be employed to mitigate HCN content and enhance food safety. Boiling is the most common household processing method used to detoxify chaya, effectively reducing cyanogenic compounds through enzymatic hydrolysis, volatilization, and leaching into the cooking medium [14,15]. However, thermal processing simultaneously induces nutrient losses, particularly for water-soluble vitamins and minerals, while exerting variable effects on bioactive compounds.

Thermal treatment is increasingly recognized as having compound-specific effects on food composition. While heat can degrade thermolabile and hydrophilic nutrients through oxidation and leaching, it may also enhance the bioaccessibility of lipophilic compounds, such as carotenoids, by disrupting cellular structures and food matrices [16,17]. This dual effect highlights a fundamental trade-off between nutrient retention, bioactive functionality, and food safety during domestic cooking.

Although previous studies have examined either cyanogenic compound reduction or phytochemical composition in chaya, comprehensive and integrative evaluations that simultaneously consider detoxification efficiency, nutrient retention, and functional bioactivity remain limited. In particular, quantitative approaches, such as the nutrient retention index (NRI), which integrate the retention of multiple nutrients into a single metric, remain underutilized in the evaluation of traditional plant foods. Existing frameworks for nutrient retention are primarily based on retention factors developed by the Food and Agriculture Organization of the United Nations/International Network of Food Data Systems and related studies, which emphasize the need for more integrative applications in real food systems [18,19].

Therefore, this study aimed to evaluate the effects of boiling on reducing cyanogenic toxicity, particularly HCN reduction, in chaya leaves while simultaneously assessing its impact on carotenoids, antioxidant capacity, and overall nutritional composition. In addition, an NRI was applied to provide an integrative assessment of nutrient preservation in relation to detoxification efficiency.

## 2. Materials and Methods

### 2.1. Chemicals and Reagents

Ethanol, hexane, and acetonitrile were purchased from RCI Labscan Limited (Bangkok, Thailand). Folin & Ciocalteu’s phenol reagent, 2,4,6-Tris(2-pyridyl)-s-triazine (TPTZ), 2,2-diphenyl-1-picrylhydrazyl (DPPH), and (±)-6-hydroxy-2,5,7,8-tetramethylchromane-2-carboxylic acid (Trolox), dipotassium hydrogen phosphate (K_2_HPO_4_), potassium dihydrogen phosphate (KH_2_PO_4_), 2′-azobis (2-amidinopropane) dihydrochloride (AAPH), and fluorescein sodium salt were purchased from Sigma-Aldrich (St. Louis, MO, USA).

For high-performance liquid chromatography (HPLC) analysis, all solvents were HPLC grade (purity ≥ 99%). Methanol, acetonitrile, and methyl tert-butyl ether (MTBE) were purchased from Fisher Scientific (Loughborough, Leicestershire, UK), and Tris was obtained from Bio Basic Inc. (Markham, ON, Canada). Standard carotenoids (lutein, zeaxanthin, and β-carotene) were purchased from Sigma-Aldrich (St. Louis, MO, USA).

### 2.2. Sample Preparation

Chaya leaves were harvested from the Ramathibodi Healthy Farm Learning Center. The dark green top 3–5 pairs of leaves were collected. The fresh chaya samples were divided into two groups: raw and boiled. For the boiled group, the leaves were immersed in boiling water at 100 °C for 15 min. The boiling conditions were selected to replicate standard household cooking practices and realistic consumption scenarios for chaya leaves. This duration of 15 min was determined based on traditional preparation recommendations, ensuring the adequate reduction of cyanogenic compounds prior to consumption. After boiling, the samples were immediately cooled in cold water for 2 min to halt further degradation. Subsequently, all samples were cut into small pieces and freeze dried until completely dry. Next, the samples were ground using a kitchen blender, vacuum-sealed in aluminum foil, and stored at −20 °C until analysis.

### 2.3. Sample Extraction

Freeze-dried fresh and boiled chaya samples were extracted as follows. One gram of dried sample was mixed with 15 mL of extraction solvent (70% ethanol or distilled water), vortex-mixed, and shaken overnight at room temperature using a wave-motion shaker (Fisherbrand™, Fisher Scientific, Pittsburgh, PA, USA). Water extraction was included to represent the aqueous fraction of bioactive compounds potentially available from boiled chaya under typical consumption conditions. The mixtures were centrifuged at 4140× *g* for 15 min using a Hettich^®^ Rotina 38R centrifuge (Andreas Hettich GmbH & Co. KG, Tuttlingen, Germany). For aqueous extraction, the supernatants were collected, designated as boiled chaya water extract (BCW), and stored at −20 °C until analysis. For ethanol extraction, the supernatants were collected, and the extraction procedure was repeated twice. The pooled extracts were concentrated under reduced pressure using a rotary evaporator (BÜCHI Labortechnik AG, Flawil, Switzerland) at 40 °C. The residues were redissolved in the corresponding solvent, transferred to amber vials, and dried under nitrogen. The resulting extracts were designated as fresh chaya ethanol extract (FCE) and boiled chaya ethanol extract (BCE) and stored at −20 °C until analysis.

### 2.4. Proximate Analysis and Mineral Content

Moisture and total ash were determined according to AOAC Official Methods 964.22 and 940.26, respectively [20]. Total fat content was determined using AOAC Official Method 922.06, and protein content (N × 6.25) was determined using AOAC Official Method 985.29 [20]. Total sugar content was quantified according to AOAC Official Method 982.14, while total carbohydrate content (including dietary fiber) was calculated based on standard nutrition labeling regulations (21 CFR Part 101) [21]. Total dietary fiber content was also determined using AOAC Official Method 985.29 [20]. Vitamins A, B1, and B2 were analyzed according to AOAC Official Methods 2011.07/2001.13, 986.27, and 985.31, respectively [20]. Mineral contents (calcium, iron, phosphorus, potassium, and sodium) were determined using AOAC Official Method 984.27 [20]. Total energy was calculated based on nutrition labeling regulations (21 CFR Part 101) [21]. Cholesterol analysis was performed according to Al-Hasani et al. [22]. Total saturated fatty acids were analyzed using AOAC Official Methods 969.33 and 996.06 [20].

### 2.5. Determination of Hydrocyanic Acid Content

The HCN content was determined directly from freeze-dried fresh and boiled chaya powders rather than from the water or ethanol extracts described in Section 2.3. The analysis was performed according to the method proposed by Kongphapa et al. [5]. Briefly, 1 g of sample was mixed with 10 mL of extraction medium consisting of 0.1 M phosphoric acid (H_3_PO_4_) containing 25% (*v*/*v*) ethanol for 2 min. The mixtures were then centrifuged at 4140× *g* for 10 min. An aliquot of 0.1 mL of each extract was mixed with 0.4 mL of 0.1 M phosphate buffer (pH 7.0), prepared from 0.1 M phosphoric acid and 0.1 M trisodium phosphate (Na_3_PO_4_). Subsequently, 0.1 mL of linamarase solution (5 U/mL, prepared in phosphate buffer, pH 6.0) was added, and the mixture was incubated at 30 °C for 15 min. After incubation, 0.6 mL of 0.2 M sodium hydroxide (NaOH) and 2.8 mL of 0.1 M phosphate buffer (pH 6.0) were added, followed by 0.2 mL of 0.5% chloramine-T solution. The mixture was incubated in an ice bath (0–4 °C) for 5 min. Subsequently, 0.8 mL of pyridine/pyrazolone solution was added, and the reaction mixture was kept at room temperature for 90 min. The absorbance was measured at 620 nm using a spectrophotometer. Potassium cyanide (KCN) was used as a standard, with concentrations ranging from 0.25 to 2.50 μg KCN (equivalent to 0.1–1.0 μg HCN). Calibration curves were constructed by plotting absorbance against KCN concentration. All analyses were performed in triplicate, and total cyanide content was expressed as mg HCN/kg fresh weight (FW).

### 2.6. Antioxidant Activity Assays

The antioxidant activity assays were performed using the 70% ethanol extracts, including fresh chaya extract (FCE) and boiled chaya extract (BCE).

#### 2.6.1. Oxygen Radical Absorbance Capacity Assay

An oxygen radical absorbance capacity (ORAC) assay, a hydrogen atom transfer (HAT)-based method, was performed as described by Huang et al. [23]. An aliquot of 25 µL of each extract, along with various concentrations of Trolox (standard) and fluorescein stock solution, was mixed with the ORAC working buffer to a final volume of 150 µL per well. The mixture was incubated at 37 °C for 30 min. Subsequently, 25 µL of AAPH solution was added to each well, and the fluorescence was immediately measured using a microplate reader equipped with a fluorescein detection system. The antioxidant capacity was expressed as micromoles of Trolox equivalents per gram of dry weight (µmol TE/g DW).

#### 2.6.2. Ferric Reducing Antioxidant Power Assay

A FRAP assay, a single electron transfer (SET)-based method, was performed as described by Panritdum et al. [24]. An aliquot of 20 µL of extract, Trolox standards, deionized water, or 70% ethanol was mixed with 150 µL of FRAP reagent and incubated at room temperature for 8 min. Absorbance was measured at 520 nm using a microplate reader (BioTek Instruments, Inc., Winooski, VT, USA). The antioxidant capacity was subsequently calculated from the Trolox calibration curve and expressed as µmol TE/g DW.

#### 2.6.3. DPPH Assay

A DPPH assay, which is a mixed-mode (hydrogen atom transfer/single electron transfer) method, was performed following Panritdum et al. [24]. An aliquot of 20 µL of extract, Trolox standards, deionized water, or 70% ethanol was mixed with 200 µL of DPPH reagent and incubated at room temperature for 30 min. Absorbance was measured at 520 nm using a microplate reader (BioTek Instruments, Inc., Winooski, VT, USA). The antioxidant capacity was subsequently calculated using the Trolox calibration curve and expressed as µmol TE/g DW.

### 2.7. Total Phenolic Content

The total phenolic content (TPC) was determined according to Panritdum et al. [24]. An aliquot of 10 μL of extract and various concentrations of gallic acid (standard) was mixed with 150 μL/well of deionized water, followed by the addition of 20 μL/well of 7.5% (*w*/*v*) NaHCO_3_ and 10 μL/well of Folin & Ciocalteu’s reagent. The mixture was incubated at room temperature for 30 min. Absorbance was measured at 750 nm using a microplate reader (BioTek^®^ Instruments, Winooski, VT, USA). The antioxidant capacity was calculated from the gallic acid calibration curve and expressed as mg gallic acid equivalents per gram of dry weight (mg GAE/g DW). The antioxidant capacity was calculated using the gallic acid (15.6–1000 mg/mL) calibration curve and expressed as mg gallic acid equivalents per gram of dry weight (mg GAE/g DW).

### 2.8. Carotenoid Analysis

#### 2.8.1. Extraction of Carotenoids

The carotenoid content was extracted following a modified version of the method proposed by Naranjo-Durán et al. [25]. Briefly, 1.0 g of freeze-dried fresh and boiled chaya was mixed with 15 mL of hexane:acetone:ethanol (2:1:1) containing 0.1% butylated hydroxytoluene (BHT) as an antioxidant. The mixture was vortexed and sonicated for 10 min, then centrifuged at 4000× *g* for 10 min at 4 °C. The supernatant was collected, and the extraction was repeated until the residue became colorless. The solvent was evaporated to dryness under a gentle stream of nitrogen at room temperature. The dried extract was reconstituted in 1 mL of methanol:methyl tert-butyl ether (MTBE) (1:1, *v*/*v*), filtered through a 0.22 µm polytetrafluoroethylene (PTFE) syringe filter, and transferred to amber HPLC vials for analysis.

#### 2.8.2. High-Performance Liquid Chromatography with Diode Array Detection Analysis

Carotenoid analysis was performed using an HPLC 1260 Infinity II system (Agilent Technologies, Santa Clara, CA, USA) equipped with a diode array detector (DAD). Separation was achieved using a C30 column (250 mm × 4.6 mm, 5 µm particle size), which allows improved resolution of carotenoid isomers. The method was adapted from Hwang et al. [26]. Carotenoids were separated at a flow rate of 1.0 mL/min with a column temperature of 30 °C using a gradient elution with two mobile phases: methanol:water (95:5, *v*/*v*) (A) and MTBE (B). The gradient elution program was as follows: 0–5 min, 95% A; 5–30 min, linear gradient to 50% A; 30–40 min, 50% A; followed by re-equilibration to the initial conditions. The column temperature was maintained at 25 °C, and the injection volume was 10 µL. Detection was carried out at 450 nm, and spectral data were recorded between 250 nm and 600 nm for compound identification. Carotenoids were identified based on retention times and UV-visible spectra compared with authentic standards. Quantification was performed using external standard calibration curves prepared from authentic carotenoid standards, including lutein, zeaxanthin, and β-carotene. Calibration curves were constructed over a concentration range of 0.1–4.5 µg/mL, and good linearity was obtained (R^2^ > 0.99). All samples were analyzed in triplicate, and results are expressed as mean ± standard deviation. Although limits of detection (LOD) and limits of quantification (LOQ) were not determined in this study, all analyte concentrations fell within the linear range of the calibration curves. To minimize carotenoid degradation, sample extracts were stored at −20 °C in amber vials and analyzed within a consistent time frame.

#### 2.8.3. Total Carotenoid Content

The total carotenoid content was determined spectrophotometrically using a modified version of the solvent extraction method proposed by Naranjo-Durán et al. [25]. Briefly, 100 mg of freeze-dried fresh and boiled chaya samples were extracted with 5 mL of cold acetone, vortex-mixed for 1 min, and incubated at 4 °C for 30 min under light-protected conditions. The mixture was then vortexed for 5 min and centrifuged at 1370× *g* for 10 min. The supernatant was collected, and the extraction was repeated three times until the residue became nearly colorless. The combined supernatants were used to measure absorbance at 450 nm using a UV-Vis spectrophotometer. The total carotenoid content was quantified using the β-carotene standard curve and expressed as mg β-carotene equivalents per 100 g fresh weight (mg BCE/100 g FW) or dry weight (mg BCE/100 g DW).

### 2.9. Statistical Analysis

All experiments were performed in triplicate and repeated in three independent experiments (*n* = 9). Results are expressed as mean ± SD. Statistical analysis was conducted using SPSS software (Version 28, IBM Corp., Armonk, NY, USA). Differences between fresh and boiled samples were evaluated using an independent samples *t*-test. A *p*-value < 0.05 was considered statistically significant. The percentage retention (%) of carotenoids after boiling was calculated based on the concentration values relative to the fresh samples using the following equation:

Retention (%) = (C_boiled_/C_fresh_) × 100

To evaluate the integrated preservation of the nutrient matrix, an NRI was derived using established nutrient retention frameworks and standardized calculation methods for cooked foods [18,27,28]. To provide a holistic assessment of nutritional integrity, the global NRI was calculated as the arithmetic mean of the retention percentages of all 12 measured parameters:

Global NRI = (1/n) × Σ(i = 1 to n) %R_i_

Furthermore, a targeted NRI for bioactives (NRI_bioactives) was formulated to reflect the stability of key functional compounds, integrating β-carotene and TPC as representative markers of lipophilic provitamin A carotenoids and hydrophilic antioxidant phenolics, respectively, and calculated as follows:

Target NRI (Bioactives) = (%R_β-carotene_ + %R_TPC_)/2

The percentage change (%) in antioxidant capacity was calculated to evaluate the effect of boiling, as follows:

%change = ((C_boiled_ − C_fresh_)/C_fresh_) × 100

where C_fresh_ and C_boiled_ represent the concentrations of carotenoids (mg/100 g) in the fresh and boiled samples, respectively.

## 3. Results

### 3.1. Nutritional Composition of Chaya Leaves

A comparative nutritional analysis of fresh and boiled chaya leaves (per 100 g) demonstrated significant differences in macronutrient, vitamin, and mineral content (Table 1). The boiling process notably influenced the caloric value and retention of water-soluble components. Boiling resulted in a substantial reduction in several key macronutrients. The protein content decreased from 7.74 ± 1.97 g in fresh leaves to 4.54 ± 0.98 g after boiling (*p* = 0.04). Similarly, total carbohydrates showed an approximately 50% reduction, decreasing from 11.20 ± 1.95 g to 5.79 ± 1.04 g (*p* = 0.03). Consequently, the overall energy value declined significantly from 85.84 ± 3.63 kcal to 53.11 ± 2.98 kcal (*p* = 0.04). While total dietary fiber and fat content varied, these changes were not statistically significant. Moreover, the boiling process had a pronounced effect on vitamin retention, with all measured vitamins indicating highly significant decreases (*p* = 0.001). The total vitamin A content decreased from 1579 ± 236 µg to 945 ± 214 µg. Thiamine (B1) was nearly entirely depleted, decreasing from 0.11 ± 0.04 mg to 0.001 ± 0.001 mg. Riboflavin (B2) also showed a considerable reduction, decreasing from 0.24 ± 0.08 mg to 0.04 ± 0.007 mg. The mineral content exhibited mixed responses to thermal treatment. Notably, potassium and iron demonstrated significant leaching, with potassium decreasing from 334 ± 96 mg to 83 ± 19 mg (*p* = 0.001) and iron from 3.85 ± 0.88 mg to 1.31 ± 0.14 mg (*p* = 0.03). Reductions in calcium (from 232 mg to 119 mg) and phosphorus (from 59.33 mg to 42.65 mg) were also recorded; however, these changes were not statistically significant (*p* = 0.07 and *p* = 0.09, respectively). Finally, the total sugar content was nearly eliminated during boiling, decreasing from 1.60 ± 0.04 g to 0.01 ± 0.001 g (*p* = 0.001).

### 3.2. Hydrocyanic Acid Content of Fresh and Boiled Chaya Leaves

**Figure 1:**
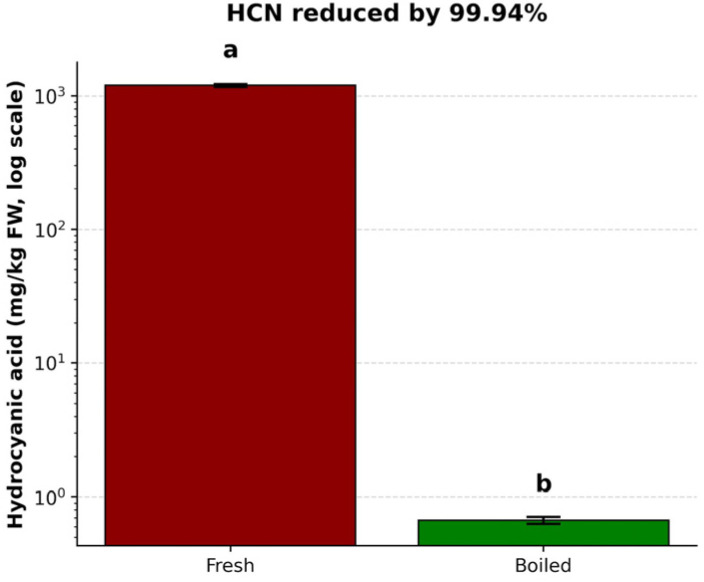
Hydrocyanic acid (HCN) content in fresh and boiled *Cnidoscolus aconitifolius* (Chaya) leaves presented on a logarithmic scale. A statistically significant difference between the two samples was identified (*p* < 0.05), as indicated by different superscript letters (a and b).

The HCN content of the chaya leaves was assessed in both fresh and boiled samples. Fresh chaya leaves contained a high mean concentration of HCN (1183.90 ± 26.60 mg/kg), a level that may pose health risks if consumed without adequate processing. In contrast, boiling reduced the HCN concentration dramatically to 0.67 ± 0.04 mg/kg, demonstrating the effectiveness of boiling as a detoxification method. To better illustrate the large difference in HCN levels between the fresh and boiled samples, the data are presented on a logarithmic scale (Figure 1). Overall, boiling achieved an approximately 99.94% reduction in HCN content relative to the fresh leaves.

Table 2 presents a summary of the reported HCN levels and the impact of thermal processing on chaya and other cyanogenic plants, as documented in previous studies. Research indicates that cooking and boiling treatments consistently lead to a reduction in residual cyanogenic content in chaya leaves compared to raw samples [5,8]. Various thermal methods evaluated by Fan & Zhou, (2010) [29], including steam heating, hot-air drying, microwave treatment, and boiling, demonstrated differing efficacy in reducing cyanogenic compounds, with boiling and microwave processing yielding the lowest levels of residual cyanogenic content. Kuti and Torres (1999) [30] reported that cooking reduced cyanogenic compounds in chaya, although quantitative HCN levels were not provided. Similarly, Ross-Ibarra and Molina-Cruz (2002) [31] highlighted boiling as a traditional preparation method used to detoxify chaya leaves prior to consumption, supporting the importance of thermal processing for improving food safety. Similar patterns have been observed in studies on cassava, where boiling and other related processing techniques significantly reduced cyanogenic compounds (Cardoso et al., 2005 [10]; Bolarinwa et al., 2016 [15]; Anorue et al., 2020 [32]).

In addition, hydrocyanic acid (HCN) does not have an established tolerable upper intake level (UL) in the same manner as nutrients. However, internationally recognized food safety guidance values are available for cyanide exposure. The Codex Alimentarius/FAO-WHO standard for edible cassava flour recommends a maximum level of 10 mg HCN/kg dry weight to minimize cyanide toxicity risk [31]. In addition, the European Food Safety Authority (EFSA) established an acute reference dose (ARfD) of 20 μg cyanide/kg body weight for dietary exposure to cyanogenic glycosides. These reference values provide an important toxicological context for evaluating the safety of residual HCN levels in boiled chaya leaves following hydrothermal processing [32].

### 3.3. Carotenoid Profiles in Fresh and Boiled Chaya Leaves

The carotenoid composition of chaya leaves was analyzed before and after boiling to assess the stability and retention of specific phytonutrients. As shown in Table 3, the compounds examined exhibited varying degrees of sensitivity to thermal processing. Lutein and zeaxanthin were significantly affected by boiling. Fresh chaya leaves contained 18.12 ± 0.60 mg/100 g lutein, which decreased to 13.85 ± 1.17 mg/100 g after boiling (*p* = 0.01), corresponding to a retention rate of 76.4%. Similarly, zeaxanthin content decreased from 0.42 ± 0.03 to 0.31 ± 0.02 mg/100 g after boiling (*p* = 0.01), with a retention rate of 73.8%, suggesting that lutein is relatively susceptible to thermal degradation and/or leaching in this plant matrix. Conversely, β-carotene exhibited exceptional stability. Its concentration decreased only slightly, from 10.43 ± 0.52 mg/100 g in fresh leaves to 9.99 ± 0.42 mg/100 g in boiled leaves. This marginal decrease was not statistically significant, resulting in a high retention rate of 95.8%. The total carotenoid content displayed a similar pattern of stability, decreasing from 31.24 ± 1.67 mg/100 g to 30.28 ± 1.16 mg/100 g after boiling. Despite the notable loss of lutein, the overall retention rate for total carotenoids remained high, at 96.9%. These findings suggest that although boiling significantly reduces lutein content, chaya leaves remain an excellent source of provitamin A (β-carotene), even after domestic cooking. The high overall retention rate underscores that thermal processing does not considerably compromise the carotenoid fraction of this vegetable.

### 3.4. Influence of Thermal Processing on Antioxidant Capacity and Total Phenolic Content

The effect of hydrothermal treatment on the bioactive potential of chaya leaves was systematically assessed using a series of antioxidant assays, including ORAC, FRAP, and DPPH, together with the measurement of the TPC. As summarized in Table 4, boiling significantly reduced antioxidant capacity across all assays (*p* < 0.05). The ORAC assay, which quantifies the ability to neutralize peroxyl radicals, revealed a 30.8% reduction, decreasing from 543.93 ± 14.87 µmol Trolox/g DW to 376.55 ± 24.66 µmol Trolox/g DW. The FRAP exhibited the greatest decline, decreasing by 49.9% from 67.27 ± 4.18 µmol Trolox/g DW to 33.69 ± 4.08 µmol Trolox/g DW, indicating a marked reduction in ferric reducing capacity. Similarly, DPPH radical scavenging activity decreased by 36.7%, reaching 10.92 ± 0.91 µmol Trolox/g DW after boiling. Additionally, the TPC, a major contributor to antioxidant activity in plant tissues, declined from 81.91 ± 9.36 GAE/g DW to 62.35 ± 6.18 mg GAE/g DW, corresponding to a 23.9% reduction.

### 3.5. Nutrient Retention

**Figure 2:**
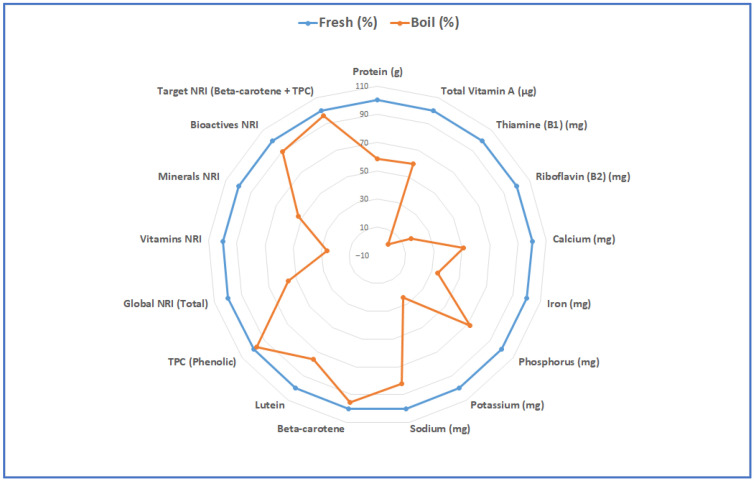
Comparative nutritional retention profile and specific retention indices (NRI) of chaya leaves following hydrothermal processing. (The radar chart illustrates the percentage retention of 12 nutritional parameters in boiled Chaya leaves, normalized against the fresh samples (represented by the outer boundary at 100%). Values are expressed as Mean ± SD (*n* = 3).

The impact of boiling on the nutritional profile of chaya leaves was further evaluated using percentage retention values and the NRI (Figure 2). β-carotene and total phenolic content (TPC) exhibited retention rates of 95.8% and 76.12%, respectively, indicating that substantial proportions of these functional compounds were preserved after boiling. Accordingly, the NRI for bioactives reached 85.95%, suggesting relatively good overall retention of representative lipophilic and hydrophilic bioactive compounds following thermal processing. Protein retention was 58.66%. Among the minerals, sodium (82.2%) and phosphorus (71.89%) showed relatively higher retention, whereas potassium retention was substantially lower at 24.85%, resulting in a mineral NRI of 52.85%. Vitamins were more affected by boiling, with total vitamin A retention of 59.85%, while riboflavin (B2) and thiamine (B1) exhibited lower retention values of 16.67% and 0.91%, respectively, resulting in a vitamin NRI of 25.81%. The overall global NRI was 54.92%. In contrast, dietary fiber and representative bioactive compounds showed comparatively higher retention following boiling.

## 4. Discussion

The present study elucidates the differential effects of boiling on the nutritional composition of chaya leaves, resulting in marked reductions in macronutrients, water-soluble vitamins, and selected minerals. These results are consistent with previous research showing that thermal processing, particularly boiling, can cause substantial nutrient losses through leaching into cooking water and thermal degradation [28,33]. The reduction in protein and carbohydrate content is likely attributable to both thermal denaturation and solubilization. Proteins can undergo structural changes during heating that increase their solubility and promote loss into the boiling medium [34]. Likewise, the significant decrease in total carbohydrates and sugars may be explained by the leaching of soluble sugars and the partial hydrolysis of polysaccharides, phenomena widely documented in leafy vegetables subjected to moist-heat treatment [35].

The notable decline in water-soluble vitamins, particularly thiamine and riboflavin, is also consistent with the established susceptibility of B vitamins to heat and aqueous loss. Thiamine, in particular, is highly heat-labile and readily degrades during cooking processes involving high temperatures and water exposure [36]. The substantial reduction in vitamin A may also suggest partial degradation and isomerization of provitamin A carotenoids, although these compounds are generally more stable than their water-soluble counterparts [37]. The mineral losses identified in this study, especially for potassium and iron, can likewise be attributed primarily to diffusion and leaching into the cooking medium. Because of its high solubility, potassium is particularly vulnerable to loss during boiling [28]. Similar trends have been observed for iron, although its retention can vary depending on food matrix interactions and cooking conditions [38]. Conversely, calcium and phosphorus showed nonsignificant reductions, likely because of their lower solubility and stronger association with the structural components of the plant tissue, which may limit their migration into the cooking water. The near-complete loss of total sugars further highlights leaching as a major mechanism of nutrient depletion during boiling and supports previous observations that simple sugars are among the first components lost during the thermal processing of leafy vegetables [33].

A major finding of the present study was the substantial reduction in HCN following boiling, demonstrating that hydrothermal processing markedly improves the safety of chaya leaves for consumption. Similar reductions in cyanogenic compounds after boiling have been reported in chaya and other cyanogenic plants [5,8,10,15,29,32], supporting thermal processing as an effective detoxification strategy. Furthermore, the residual HCN levels observed after boiling were substantially lower than internationally recognized cyanide safety guidance values, including the FAO/WHO recommended maximum level of 10 mg HCN/kg dry weight for cyanogenic foods [39] and the EFSA acute reference dose of 20 μg cyanide/kg body weight [40]. However, insufficiently processed or raw chaya leaves may still pose potential cyanogenic toxicity risks, emphasizing the importance of adequate cooking prior to consumption.

The detoxification effect of boiling is primarily explained by the enzymatic hydrolysis of cyanogenic glycosides following tissue disruption, which releases HCN that can subsequently be removed through volatilization and leaching during thermal processing [10,14]. The relatively high initial HCN concentration observed in fresh chaya leaves (1183.90 mg/kg) suggests considerable toxicological concern if consumed without processing, whereas the residual concentration after boiling (0.67 mg/kg) indicates highly effective detoxification. Collectively, these findings support boiling as a practical household preparation method for reducing cyanogenic risk in chaya leaves.

Among the evaluated bioactive compounds, carotenoids exhibited relatively high thermal stability, with β-carotene retention reaching 95.8%. This stability may be related to the lipophilic nature of β-carotene, its localization within chloroplast membranes, and its relative resistance to thermal degradation under moderate heating conditions [16,17]. Conversely, lutein showed greater susceptibility to processing, which may be associated with its more polar structure and higher sensitivity to isomerization and oxidative degradation [41,42]. These findings highlight the importance of compound-specific evaluation, as total carotenoid measurements alone may not fully capture compositional changes during thermal processing.

In contrast to carotenoids, phenolic compounds and antioxidant capacity were more susceptible to thermal processing. Boiling caused significant reductions in total phenolic content and antioxidant capacity, as evidenced by the ORAC, FRAP, and DPPH assays. These decreases may be associated with thermal degradation, oxidative reactions, and leaching of water-soluble phenolics during boiling [43,44,45]. The greater reduction observed in FRAP suggests preferential losses of electron-donating antioxidants, including low-molecular-weight phenolics and ascorbic acid [46,47]. However, these in vitro findings should be interpreted cautiously because thermal disruption of the plant matrix may influence the release and bioaccessibility of bound phenolic compounds [48,49].

Furthermore, the proposed NRI provided an integrative framework for evaluating the multidimensional effects of thermal processing and revealed distinct differences among nutrient classes. Representative bioactive compounds showed relatively high retention (NRI = 85.96%), whereas vitamins (25.81%) and minerals (52.85%) were more susceptible to processing losses, resulting in a moderate global NRI of 54.92%. This pattern of differential retention aligns with previous studies showing nutrient-specific responses to thermal processing [36,45] and with established nutrient retention models [18,27]. However, prior work has largely examined nutrient degradation and detoxification in isolation. In contrast, the present study integrates these dimensions and reveals a critical but underexplored trade-off between micronutrient preservation and toxicological safety. In this context, boiling emerges as a dual-function process that effectively eliminates cyanogenic compounds while selectively reshaping the nutrient matrix. Notably, the preferential retention of carotenoids, dietary fiber, and phenolic bioactives further underscores the resilience of functionally relevant compounds and suggests that the health-promoting potential of boiled chaya is maintained despite the loss of more labile micronutrients. Taken together, these findings support the use of the NRI as a translational metric linking nutrient retention with detoxification efficiency and provide a mechanistic basis for optimizing processing strategies at the interface of food toxicology and nutritional science.

A key strength and innovative aspect of this study involves in its integrative analytical framework, which concurrently assessed cyanogenic detoxification, carotenoid stability, and antioxidant capacity through multiple assays, as well as the Nutrient Retention Index (NRI) in the hydrothermal processing of chaya leaves. In contrast to previous studies that primarily concentrated on the reduction of cyanogenic compounds, this multidimensional approach provides a more comprehensive understanding of the equilibrium between food safety and nutritional quality in the edible form that is commonly consumed. Additionally, the inclusion of NRI analysis provides valuable insights into the nutritional contribution and retention of bioactive compounds following boiling, thereby enhancing the dietary relevance of the findings.

Several limitations must be acknowledged in this study. First, our research primarily employed in vitro analytical approaches, which may not intensively represent in vivo bioavailability, metabolic transformation, or physiological responses to the retained bioactive compounds. Second, the study evaluated only a single boiling condition—100 °C for 15 min—which was chosen to reflect a standardized and realistic household preparation method commonly recommended for consuming chaya and detoxifying cyanogenic compounds. As a result, the findings are specific to the processing condition investigated, rather than reflecting optimized cooking processes. Future research assessing different cooking durations and methods may provide further insights into the balance between nutrient retention and the removal of hazardous compounds. Third, the boiling water was not analyzed, which limited our ability to evaluate mass balance and quantify nutrient migration during the processing. The observed reductions in HCN, vitamins, minerals, carotenoids, phenolics, and antioxidant capacity were interpreted based on known physicochemical properties, including the water solubility and volatility of HCN, as well as the susceptibility of water-soluble compounds to leaching and thermal degradation. Therefore, the proposed mechanisms remain partially inferential. Future studies that include an analysis of the cooking water may offer a more comprehensive understanding of nutrient migration and the distribution of cyanogenic compounds during thermal processing. Finally, we intentionally expressed nutritional composition and carotenoid data on a wet weight basis to reflect the forms commonly consumed by Thai populations and to enhance practical dietary relevance. However, differences in moisture content between fresh and boiled samples might affect direct compositional comparisons. To facilitate more standardized comparisons, future investigations should also evaluate nutrient retention based on dry weight (dwb).

The current findings offer valuable insights into nutrient retention and detoxification during boiling food at home. However, further research is necessary to fully understand the nutritional and toxicological effects of thermal processing. Future studies should combine both in vitro and in vivo methods, including simulated gastrointestinal digestion models and studies involving animals or humans, to better assess the bioavailability and physiological significance of retained nutrients and bioactive compounds. Additionally, comparing various cooking methods and processing conditions could enhance our understanding of the balance between preserving nutrients and detoxifying harmful compounds. Furthermore, integrating toxicokinetic assessments with nutrient retention analyses may create a more comprehensive framework for optimizing food processing strategies, addressing the interconnected areas of nutrition, food safety, and toxicology.

## 5. Conclusions

Boiling chaya produces selective and compound-specific changes that significantly reduce the levels of water-soluble micronutrients while largely preserving lipophilic carotenoids, such as β-carotene. Notably, this process achieves near-complete elimination of HCN, thus underscoring its critical role in ensuring food safety. The proposed NRI reveals a distinct processing trade-off between micronutrient loss and toxicological risk reduction. Rather than functioning solely as a degradative process, boiling acts as a targeted transformation that modifies the nutrient matrix while maintaining the edibility of the leaves. Although labile nutrients are lost, the preservation of provitamin A carotenoids and other essential bioactives helps sustain the functional value of boiled chaya. Overall, these findings establish an integrative framework that links nutrient retention with detoxification efficiency and offer a scientific foundation for optimizing household processing strategies at the intersection of food toxicology, functional foods, and public health nutrition.

## Figures and Tables

**Table 1:** Nutritional Composition of Fresh and Boiled Chaya Leaves (100 g).

Test Items	Fresh Chaya Leaves (*n* = 3)	Boiled Chaya Leaves (*n* = 3)	*p*-Value
Moisture, g	77.97 ± 10.36	87.77 ± 11.87	0.42
Ash, g	1.97 ± 0.003	0.59 ± 0.004	0.08
Protein (N × 6.25),	7.74 ± 1.97	4.54 ± 0.98	0.04
Total fat, g	1.12 ± 0.05	1.31 ± 0.07	0.27
Total carbohydrate, g (Included fiber)	11.20 ± 1.95	5.79 ± 1.04	0.03
Total dietary fiber, g	9.15 ± 1.76	5.01 ± 1.26	0.11
Energy from fat, Kilocalories	10.08 ± 2.47	11.79 ± 1.09	0.24
Total energy, Kilocalories	85.84 ± 3.63	53.11 ± 2.98	0.04
Total vitamin A, µg	1579 ± 236	945 ± 214	0.001
Thiamine, mg	0.11 ± 0.04	0.001 ± 0.001	0.001
Riboflavin, mg	0.24 ± 0.08	0.04 ± 0.007	0.001
Calcium, mg	232 ± 69	119 ± 31	0.07
Iron, mg	3.85 ± 0.88	1.31 ± 0.14	0.03
Phosphorus, mg	59.33 ± 6.65	42.65 ± 4.69	0.09
Potassium, mg	334 ± 96	83 ± 19	0.001
Sodium, mg	10.0 ± 2.03	8.22 ± 1.09	0.14
Total sugar, g	1.60 ± 0.04	0.01 ± 0.001	0.001
Total saturated fatty acid, g	0.30 ± 0.08	0.40 ± 0.09	0.06
Cholesterol, mg	0.01 ± 0.004	0.01 ± 0.009	0.84

**Table 2:** Comparison of Hydrocyanic Acid (HCN) Levels and Reduction after Processing in Chaya and Other Cyanogenic Plants.

Study	Plant	Processing	HCN (mg/kg FW)	Reduction (%)	Remark
This study	*Cnidoscolus aconitifolius*	Fresh	1183.90 ± 26.60	-	High HCN
		Boiled	0.67 ± 0.04	99.94%	Substantial reduction
Kongphapa et al. (2021) [5]	*Cnidoscolus chayamansa* Mc. Vaugh	Raw	112.272 ± 22.46	98.48%	Substantial reduction
		Cooked	1.71 ± 0.04		
Kuri-García et al. (2017) [8]	Chaya	Raw	23.7–42.5	-	Cyanogenic compounds present
		Boiled (5 min)	Not detected	~100%	Boiling eliminates cyanide
Fan & Zhou, (2010) [29]	Chaya	Raw	Not specified	-	Cyanogenic glycosides as major anti-nutritional factor
		Steam heating	31.31% remaining	68.69%	Partial reduction
		Hot-air drying	40.69% remaining	59.31%	Moderate reduction
		Microwave	3.74% remaining	96.26%	High reduction
		Boiling	2.17% remaining	97.83%	Most effective
		Microwave/Boiling	<5 mg/kg	-	Meets safety level
Kuti & Torres (1999) [30]	Chaya	Cooked	Not quantified	-	Cyanogenic compounds reduced
Ross-Ibarra & Molina-Cruz (2002) [31]	Chaya	Boiled	Not quantified	-	Traditional detoxification
Cardoso et al. (2005) [10]	Cassava	Boiled/processed	~10–50	>90%	Effective cyanogen removal
Bolarinwa et al. (2016) [15]	Cassava	Boiled/fermented	5–20	>90%	Significant detoxification
Anorue et al. (2020) [32]	Cassava	Boiling/drying	<10	>90%	Safe consumption level

**Table 3:** Carotenoid Profiles and Retention (%) in Fresh and Boiled Chaya Leaves.

	Fresh Chaya (mg/100 g Fresh Weight)	Boiled Chaya (mg/100 g Fresh Weight)	Retention (%)
Lutein	18.12 ± 0.60	13.85 ± 1.17 *	76.4
Zeaxanthin	0.42 ± 0.03	0.31 ± 0.02 *	73.8
Beta-carotene	10.43 ± 0.52	9.99 ± 0.42	95.8
Total carotenoids	31.24 ± 1.67	30.28 ± 1.16	96.9

* Significant difference from fresh Chaya, *p* = 0.01.

**Table 4:** Antioxidant Capacities (ORAC, FRAP, DPPH, and TPC) and Percentage Change (%) in Fresh and Boiled Thai Edible Leaves.

Group	ORAC (µmol Trolox /g DW)	FRAP (µmol Trolox /g DW)	DPPH (µmol Trolox /gDW)	Total Polyphenol Content (mgGAE/gDW)
Fresh Chaya	543.93 ± 14.87 ^a^	67.27 ± 4.18 ^a^	17.25 ± 2.25 ^a^	81.91 ± 9.36 ^a^
Boiled Chaya	376.55 ± 24.66 ^b^	33.69 ± 4.08 ^b^	10.92 ± 0.91 ^b^	62.35 ± 6.18 ^b^
% Change	−30.8	−49.9	−36.7	−23.9

Values are expressed as mean ± SD (*n* = 3). Different superscript letters within the same column indicate significant differences at *p* < 0.05.

## Data Availability

The original contributions presented in the study are included in the article, further inquiries can be directed to the corresponding author.
